# Generalized analytic formula for spin Hall effect of light: shift enhancement and interface independence

**DOI:** 10.1515/nanoph-2021-0794

**Published:** 2022-01-26

**Authors:** Minkyung Kim, Dasol Lee, Yeseul Kim, Junsuk Rho

**Affiliations:** Department of Mechanical Engineering, Pohang University of Science and Technology (POSTECH), Pohang 37673, Republic of Korea; Department of Biomedical Engineering, Yonsei University, Wonju 26493, Republic of Korea; Department of Chemical Engineering, Pohang University of Science and Technology (POSTECH), Pohang 37673, Republic of Korea; POSCO-POSTECH-RIST Convergence Research Center for Flat Optics and Metaphotonics, Pohang 37673, Republic of Korea

**Keywords:** arbitrary polarization, elliptical polarization, optical spin Hall effect, photonic spin Hall effect, spin Hall shift

## Abstract

The spin Hall effect of light (SHEL) is the microscopic spin-dependent splitting of light at an optical interface. Whereas the spin Hall shift under linearly polarized light is well-formulated, studies on the SHEL under elliptically or circularly polarized light have primarily relied on numerical computation. In this work, an explicit analytic formula for the spin Hall shift is derived under arbitrarily polarized incidence. Furthermore, from this explicit expression, we demonstrate that the spin Hall shift can be enhanced at any incident angle by using polarization degree of freedom and is independent of the Fresnel coefficients of an interface under circularly polarized light. The analytic formula will help us understand the SHEL under general polarization intuitively and realize unprecedented modulation of the SHEL.

## 1 Introduction

The light that is reflected or refracted at an optical interface experiences a microscopic spin-dependent spatial displacement along the perpendicular direction [1, 2]. This natural phenomenon arises from the vectorial and transversal nature of light and forms an optical analogy to the spin Hall effect [3, 4] and thus is referred to as the spin Hall effect of light (SHEL) [5]. The SHEL has been intensively studied [6–8] at various interfaces such as interfaces between natural materials [9–12] and the boundaries of artificial media [13–22]. Linearly polarized light is split in half into two circularly polarized components with opposite handedness by the same displacement but in opposite directions [9].

This symmetrical splitting in shift and intensity, however, occurs only under linearly polarized incidence at an interface whose eigenmodes are linearly polarized. Otherwise, i.e., if the incidence is elliptically or circularly polarized and/or the interface does not preserve the polarization states of *s*- or *p*-polarized incidence, the splitting is no longer symmetrical [23–26]. In such cases, the magnitudes of the spin Hall shift of left circularly polarized (LCP) and right circularly polarized (RCP) components are generally not equal. In contrast to the symmetrical SHEL under horizontally polarized incidence, the SHEL under a more general polarization, i.e., that under elliptical or circular polarizations, has been less explored [23, 27, 28]. Recently, a full-wave theory of the SHEL has been established in a circular basis, expediting the rigorous understanding of the SHEL in the broader context [26, 29, 30]. However, in the study of the SHEL under arbitrarily polarized incidence, calculation of the spin Hall shift under those incidences have relied on numerical computation by taking the *y* position average of the spatial field profiles of reflected beams. However, an analytic formula for the spin Hall shift may provide better insights into the SHEL, enabling us to understand the SHEL under general incidence intuitively and straightforwardly and thus opening a route to control the SHEL more diversely.

Here, we derive an explicit analytic formula for the spin Hall shift under arbitrarily polarized incidence and explain how this formula can enrich SHEL studies. As an example, we use the formula to demonstrate that the SHEL can be enhanced at an arbitrary incident angle by modulating the incident polarization state and that interface dependence, which has been considered as an intrinsic attribute of the SHEL, disappears under circularly polarized incidence. We believe that the analytic expression of the spin Hall shift under arbitrarily polarized incidence would be a great starting point to reveal numerous fascinating spin Hall-related phenomena in addition to the two specific examples considered in this work and will bring out interesting follow-up studies.

## 2 Formula for spin Hall shift under arbitrarily polarized incidence

**Figure 1:**
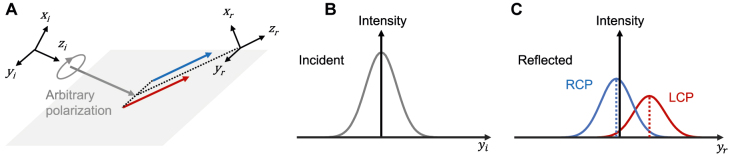
Schematic of the SHEL. (A) Beam reflection at a planar interface. (B) and (C) Beam profiles of (B) incident and (C) reflected beams.

This section describes the derivation of the explicit analytic formula for the spin Hall shift under arbitrarily polarized incidence. We focus on the SHEL of reflected beams (Figure 1); however, this study can be straightforwardly extended to refracted beams. Here, we restrict our scope to the interfaces that have no cross-polarization Fresnel coefficients (*r*_*sp*_ = *r*_*ps*_ = 0). An incident Gaussian beam in spatial coordinates $\left(\begin{array}{c} x_{i},\;y_{i},\;z_{i} \end{array}\right)$ (see Figure 1A for the definition of the coordinates) can be expressed as a product of a Jones vector corresponding to the polarization state and a scalar function representing the Gaussian beam shape, as follows:

(1) $$\psi_{i}=\left(\begin{array}{c} \psi_{\text{H}}\\ \psi_{\text{V}} \end{array}\right)\exp\left(-k_{0}\frac{x_{i}^{2}+y_{i}^{2}}{z_{\text{R}}+iz_{i}}+ik_{0}z_{i}\right),$$

where *ψ*_H_ and *ψ*_V_ are complex elements of the Jones vector corresponding to horizontal and vertical polarizations, respectively, *k*_0_ is the wave vector in the medium, and $z_{\text{R}}=k_{0}w_{0}^{2}/2$ is a Rayleigh length for beam waist *w*_0_. The beam reflected at an interface with Fresnel reflection coefficients *r*_*s*_ and *r*_*p*_ follows [31]

(2) $$\begin{array}{ll} \psi_{r} & =\left(\begin{array}{c} \psi_{\text{H}}r_{p}\left(1-i\frac{x_{r}}{z_{\text{R}}+iz_{r}}\frac{\partial \ln r_{p}}{\partial \theta_{i}}\right)+i\psi_{\text{V}}\frac{y_{r}}{z_{\text{R}}+iz_{r}}\left(r_{p}+r_{s}\right)\cot\theta_{i}\\ \psi_{\text{V}}r_{s}\left(1-i\frac{x_{r}}{z_{\text{R}}+iz_{r}}\frac{\partial \ln r_{s}}{\partial \theta_{i}}\right)-i\psi_{\text{H}}\frac{y_{r}}{z_{\text{R}}+iz_{r}}\left(r_{p}+r_{s}\right)\cot\theta_{i} \end{array}\right)\exp\left(-k_{0}\frac{x_{r}^{2}+y_{r}^{2}}{z_{\text{R}}+iz_{r}}+ik_{0}z_{r}\right), \end{array}$$

where $\left(\begin{array}{c} x_{\text{r}},\;y_{\text{r}},\;z_{\text{r}} \end{array}\right)$ is the coordinate of the reflected beam and *θ*_*i*_ is the incident angle. To derive an analytic expression for the spin Hall shift, we examine the circularly polarized component of the reflected beam, $\psi_{\text{r}}^{\pm }=e^{\mp }\cdot \psi_{\text{r}}$ ( + for LCP and − for RCP), for $e^{\pm }={\left(\begin{array}{c} 1,\;\pm i \end{array}\right)}^{\text{T}}/\sqrt{2}$ . Then, at *x*_r_ = 0, we use the first-order Taylor expansion, 1 + *α* ≈ exp(*α*), to approximate the two circularly polarized reflected beams as

(3) $$\begin{array}{ll} \psi_{\text{r}}^{\pm }\left(x_{\text{r}}=0\right) & \propto \left(\psi_{\text{H}}r_{p}\mp i\psi_{\text{V}}r_{s}\right)\exp\left[-\frac{k_{0}}{2\left(z_{\text{R}}+iz_{\text{r}}\right)}\right.\\ & \left.\;\times {\left(y_{\text{r}}\pm \frac{\psi_{\text{H}}\mp i\psi_{\text{V}}}{\psi_{\text{H}}r_{p}\mp i\psi_{\text{V}}r_{s}}\frac{r_{p}+r_{s}}{k_{0}}\cot\theta_{i}\right)}^{2}\right], \end{array}$$

where the first term on the right-hand side corresponds to the amplitude and the second term denotes the Gaussian beam shape with spatial displacement along the *y*-axis. Equation (3) clearly demonstrates that the beam is shifted by

(4) $$\delta^{\pm }=\mp \text{Re}\left(\frac{\psi_{\text{H}}\mp i\psi_{\text{V}}}{\psi_{\text{H}}r_{p}\mp i\psi_{\text{V}}r_{s}}\frac{r_{p}+r_{s}}{k_{0}}\cot\theta_{i}\right),$$

along the *y*-axis. This equation explicitly represents the spin Hall shift under arbitrarily polarized incidence. Let us now discuss the implication of Eq. (4). Imaginary unit *i* in Eq. (4) is attributed to the *π*/2 phase rotation in a complex plane that occurs during the transformation from the linear basis to the circular basis. The first fraction inside the parentheses represents a circularly polarized component of the incidence divided by the identical polarization component of the reflected beam. In other words, the spin Hall shift is directly proportional to the projection of the ratio between the circularly polarized components of the incident and reflected beams to the real axis.

Equation (4) is simpler and more intuitive than the analytic formula for the spin Hall shift under arbitrarily polarized incidence presented in the supporting material of ref. [9]. By directly obtaining the simplified form of the analytic expression from the beam profile directly and retaining the complex quantity, we can extract interesting information from Eq. (4). For example, Eq. (4) explicitly proves the recent proposal that an interface with *r*_*s*_ = *r*_*p*_ supports the incident-polarization-independent SHEL [28]. The substitution of *r*_*s*_ = *r*_*p*_ ≡ *r* into Eq. (4) eliminates *ψ*_H_ and *ψ*_V_ and gives *δ* ^±^ = ∓2 cot *θ*_*i*_/*k*_0_. In addition, Eq. (4) reproduces the previously reported expressions for the spin Hall shift under horizontally (*ψ*_H_ = 1, *ψ*_V_ = 0) and vertically (*ψ*_H_ = 0, *ψ*_V_ = 1) polarized incidences, which are given by [10]

(5) $$\begin{array}{ll} \delta_{\text{H}}^{\pm } & =\mp \frac{\cot\;\theta_{i}}{k_{0}}\text{Re}\left(1+\frac{r_{s}}{r_{p}}\right),\\ \delta_{\text{V}}^{\pm } & =\mp \frac{\cot\;\theta_{i}}{k_{0}}\text{Re}\left(1+\frac{r_{p}}{r_{s}}\right). \end{array}$$

Note that the assumption underlying Eq. (4) and consequentially Eq. (5) is that the spin Hall shift is considerably smaller than the beam waist $\left(|\delta^{\pm }|\ll w_{0}\right)$ so that ${\left(k_{0}w_{0}\right)}^{2}\gg \cot^{2}\;\theta_{i}$ is satisfied. Then, the higher-order terms in the Taylor expansion, which are on the order of *O* ^2^(*δ*/*w*_0_) and beyond, can be neglected. Such a condition generally holds, except in the case of a tightly-focused beam under near-normal incidence, i.e., when *w*_0_ is not sufficiently larger than |*δ* ^±^| and *θ*_*i*_ ≪ 1° at the same time.

**Figure 2:**
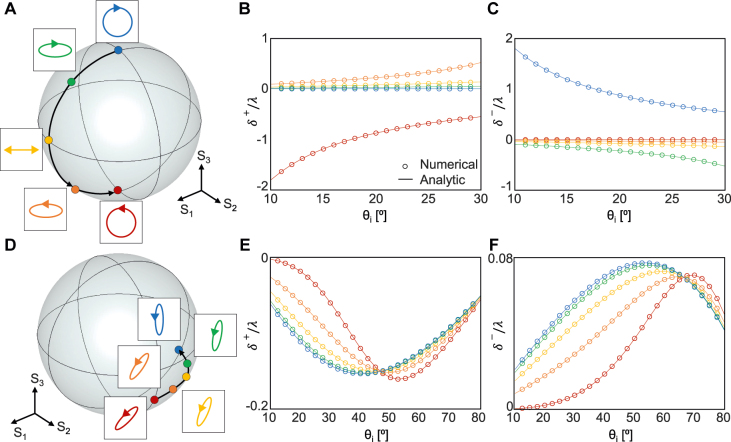
Spin Hall shift at an air–BK7 interface (*n*_1_ = 1, *n*_2_ = 1.515) under various incident polarization states. (A) Poincaré sphere with five incident polarization states with *S*_2_ = 0. (B) *δ* ^+^/*λ* and (C) *δ* ^−^/*λ* of the incidences. (D) Poincaré sphere with five incident polarization states with *S*_3_ = −0.5. (E) *δ* ^+^/*λ* and (F) *δ* ^−^/*λ* of the incidences. The colors of curves (analytic) and markers (numerical) in (B), (C), (E) and (F) are identical to those of the markers in (A) and (D).

To ensure the reliability of Eq. (4), *δ* ^±^/*λ* under 10 incidences with different polarization states are examined where *λ* is the wavelength (Figure 2). The Stokes parameters, $\left(\begin{array}{c} S_{1},\;S_{2},\;S_{3} \end{array}\right)$ , of five distinct incident polarizations are represented in the Poincaré sphere by fixing *S*_2_ (Figure 2A) and *S*_3_ (Figure 2D). In each case, the spin Hall shift is calculated using the analytic formula (Eq. (4), curves in Figure 2) and then compared with the numerical value (markers) that is directly obtained using the *y* position average as

(6) $$\delta^{\pm }=\text{Re}\frac{\left\langle {\tilde{\psi }}_{\text{r}}^{\pm }|y_{\text{r}}|{\tilde{\psi }}_{\text{r}}^{\pm }\right\rangle }{\left\langle {\tilde{\psi }}_{\text{r}}^{\pm }|{\tilde{\psi }}_{\text{r}}^{\pm }\right\rangle },$$

where ${\tilde{\psi }}_{\text{r}}$ is the spatial profile of the reflected beam, whose expression can be obtained by the Fourier transform of *ψ*_r_ or from other literature [11]. We use *w*_0_ = 10^3^ *λ* and *z*_r_ = 10*λ* in the numerical calculation. The analytic and numerical results perfectly match each other, thereby confirming that Eq. (4) is credible. In the following sections, we use this formula to demonstrate two features, (i) the enhancement of the SHEL via polarization control and (ii) interface-independent SHEL under circular polarization.

## 3 Enhancement of spin Hall shift via polarization control

Equation (4) reveals the relationship between the incident polarization and spin Hall shift explicitly. Therefore, we can obtain an explicit expression of an incident polarization state for which the spin Hall shift diverges. For an interface whose reflection coefficients are *r*_*s*_ and *r*_*p*_, an incident polarization of

(7) $$\left(\begin{array}{c} \psi_{\text{H}}\\ \psi_{\text{V}} \end{array}\right)=\left(\begin{array}{cc} \cos△ & -\sin△\\ \sin△ & \cos△ \end{array}\right)\frac{1}{\sqrt{|r_{s}{|}^{2}+|r_{p}{|}^{2}}}\left(\begin{array}{c} \pm ir_{s}\\ r_{p} \end{array}\right),$$

for sufficiently small △ (≪1 rad) significantly increases *δ* ^±^. Here, the vector on the right-hand side is defined to make the denominator of Eq. (4) (*ψ*_H_ *r*_*p*_ ∓ *iψ*_V_ *r*_*s*_) zero, and the matrix corresponds to an infinitesimal rotation to prevent Eq. (4) from being singular. Therefore, by substituting the Jones vector from Eq. (7) into Eq. (4), it can be demonstrated that *δ* ^±^ increases beyond the wavelength scale around *θ*_*i*_ where *r*_*s*_ and *r*_*p*_ are defined.

**Figure 3:**
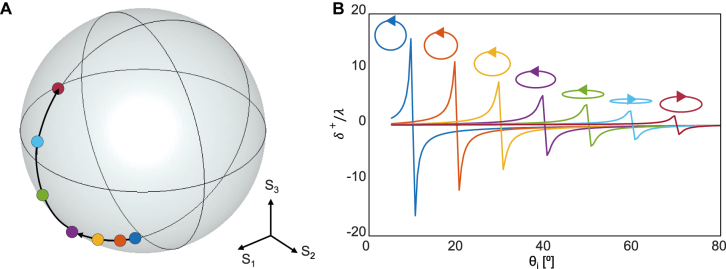
Enhancement of the spin Hall shift at an air–BK7 interface via polarization control. (A) Poincaré sphere with seven incident polarization states and (B) *δ* ^+^/*λ* under the incidences. The seven polarization states are selected to enhance *δ* ^+^/*λ* at *θ*_*i*_ ranging from 10° to 70° in steps of 10° using Eq. (7) with △ = 1°.

Figure 3 demonstrates that *δ* ^+^/*λ* can be significantly increased with sharp peaks for *θ*_*i*_ ranging from 10° to 70° in steps of 10° by varying the incident polarization state using Eq. (7) with △ = 1°. The maximum value of *δ* ^+^/*λ* decreases as *θ*_*i*_ increases because of cot *θ*_*i*_ in Eq. (4) and can be further enhanced by reducing △. However, if △ is too small that *δ* ^+^/*λ* becomes sufficiently large to make the higher-order terms (*O* ^2^(*δ*/*w*_0_)) no longer negligible, Eq. (4) deviates from the numerical values obtained by direct averaging. It should be noted that the analytic results shown in Figure 3 agree well with the numerical values in our parameter space.

This strategy is similar to the enhancement of the SHEL under horizontally polarized incidence close to the Brewster angle [10], [11], [12, 32], [33], [34]. Indeed, the SHEL near the Brewster angle, which is approximately 56.6° in this specific example, can be understood as a subset of this approach where the incidence has *S*_3_ = 0 (Figure 3A, between the light blue and green markers). The underpinning of the spin Hall shift amplification is similar to that near the Brewster angle [29] in that the intensity of incidence that approaches zero enforces the divergence of the geometric phase underlying the SHEL. The geometric phase originates from the noncentral wave vectors that are not parallel to the central propagation direction and the consequent polarization basis rotation in the three-dimensional momentum space. Near the target incident angle, the reflection coefficient of the incident central wave vector converges to zero, but that of the noncentral wave vector remains finite. The Berry phase singularity arising from this infinite relative strength results in the remarkably increased spin Hall shift.

In comparison to the SHEL enhancement near the Brewster angle, this approach has a clear advantage that the value of *θ*_*i*_ at which the SHEL is enhanced can be tuned by adjusting the incident polarization. Whereas the SHEL enhancement near the Brewster angle is only achievable at a specific angle, *θ*_B_ = atan(*n*_2_/*n*_1_), which is exclusively determined by the refractive indices of the media (*n*_1_ and *n*_2_), this method provides the enhancement of the SHEL at any target *θ*_*i*_ by substituting *r*_*s*_ and *r*_*p*_ at that *θ*_*i*_ into Eq. (7). One disadvantage of this method is that *δ* ^+^/*λ* and *δ* ^−^/*λ* are asymmetric yet coupled; hence, they cannot be independently enhanced or controlled. Nevertheless, this approach provides a viable route to enhance and/or control the spin Hall shift using the polarization degree of freedom.

## 4 Interface-independent spin Hall shift under circularly polarized incidence

Furthermore, Eq. (4) demonstrates straightforwardly that the spin Hall shift under circularly polarized incidence is independent of the interface, i.e., insensitive to *r*_*s*_ and *r*_*p*_. The substitution of $\psi_{\text{H}}=1/\sqrt{2}$ and $\psi_{\text{V}}=\pm i/\sqrt{2}$ into Eq. (4) gives *δ* ^±^ = ∓2 cot *θ*_*i*_/*k*_0_ for the same handedness, as the sum of the reflection coefficients is canceled out, and zero spin Hall shift for the opposite case. Note that here ± in *ψ*_V_ and that in *δ* are independent. This interface independence under circular polarization that contradicts the conventional idea that the SHEL is inevitably interface dependent has been recently mentioned in ref. [29], where the spin Hall shift is obtained by taking *k*_*y*_-derivative of the Pancharatnam–Berry phase. Here, based on Eq. (4), we show that whereas the handedness flip during the reflection at an isotropic-isotropic interface leads to the interface-independent spin Hall shift with near-zero efficiency, the gigantic and interface-independent spin Hall shift can be achieved with relatively high efficiency at an isotropic–anisotropic interface that preserves the handedness.

**Figure 4:**
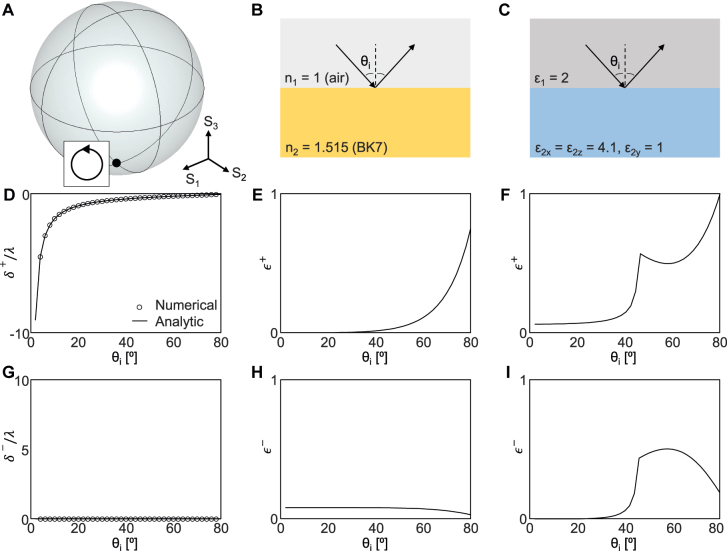
Interface-independent spin Hall shift under LCP incidence. (A) Polarization state of the incidence represented in Poincaré sphere. (B) and (C) Schematics of reflection at (B) air–BK7 interface and (C) isotropic–anisotropic (*ɛ*_1_ = 2, *ɛ*_2_ = diag(4.1, 1, 4.1)) interface. (D) *δ* ^+^/*λ* and (G) *δ* ^−^/*λ* at two interfaces. (E) *ϵ* ^+^ and (H) *ϵ* ^−^ at the air–BK7 interface. (F) *ϵ* ^+^ and (I) *ϵ* ^−^ at the isotropic–anisotropic interface.

For numerical confirmation, we consider the reflection of the LCP ( $\psi_{\text{H}}=1/\sqrt{2}$ and $\psi_{\text{V}}=i/\sqrt{2}$ ) incidence at two interfaces (Figure 4), one between two isotropic media (Figure 4B) and the other between isotropic and anisotropic media (Figure 4C). The former is introduced as a general example, and the latter is to provide *π* phase delay between two linear polarizations under normal incidence [28]. As expected from Eq. (4), *δ* ^+^/*λ* = −2 cot *θ*_*i*_/*k*_0_ (Figure 4D) and *δ* ^−^/*λ* = 0 (Figure 4G). These two distinct interfaces exhibit the same spin Hall shift, confirming that the SHEL is indeed independent of the interface. Note that the nonzero spin Hall shift does not diverge to infinity as *θ*_*i*_ approaches zero (Figure 4D) but is bounded to *w*_0_/2 because Eq. (4) is invalidated if *θ*_*i*_ is sufficiently small (≪1°).

The efficiencies, *ϵ* ^±^ = |*ψ*_H_ *r*_*p*_ ∓ *iψ*_V_ *r*_*s*_|^2^, which are defined as the intensities of the corresponding polarizations, are interface dependent. Here, we focus only on small values of *θ*_*i*_, for which *δ* ^+^/*λ* has a large magnitude. Within this range, the reflected beam has dominant opposite polarization (Figure 4E and H) at the interface between two isotropic media (Figure 4B). In other words, most of the reflected beam is the RCP component with zero spin Hall shift (Figure 4G and H), whereas the LCP component with large spin Hall shift has a negligible intensity (Figure 4D and E).

This issue can be overcome by adopting an isotropic–anisotropic interface (Figure 4C). Here, the permittivity of the anisotropic medium along the perpendicular direction is smaller than that of the isotropic medium while the permittivities along the remaining directions are larger (*ɛ*_2*y*_ < *ɛ*_1_ < *ɛ*_2*x*_, *ɛ*_2*z*_), leading to phase shift *π* between *r*_*s*_ and *r*_*p*_. This sign change retains *δ* ^±^/*λ* unaltered (Figure 4D and G) but reverses the handedness of the reflected beam and therefore notably enhances the reflection of the co-polarized reflected beam (Figure 4F) at the expense of cross-polarized reflection (Figure 4I). In short, the SHEL under circular polarization is insensitive to the reflection coefficients and the efficiency of the reflected beam with the large spin Hall shift can be enhanced by adopting an isotropic–anisotropic interface with appropriate permittivity relations.

## 5 Conclusions

An explicit analytic formula for the spin Hall shift under arbitrarily polarized incidence is developed. Then, on the basis of the analytic expression, we propose a way to enhance the SHEL at an arbitrary incident angle via incident polarization control and demonstrate that the large and interface-independent spin Hall shift can be observed under circularly polarized incidence if the handedness is preserved during the reflection by using an isotropic–anisotropic interface. The explicit formula for the spin Hall shift under general polarization will enable us to intuitively understand and predict numerous fascinating SHEL and enrich future research.
